# Disease Modifying Therapy in Multiple Sclerosis

**DOI:** 10.1155/2014/307064

**Published:** 2014-07-07

**Authors:** U. E. Williams, S. K. Oparah, E. E. Philip-Ephraim

**Affiliations:** Internal Medicine Department, University of Calabar, Calabar 540242, Cross River State, Nigeria

## Abstract

Multiple sclerosis is an autoimmune disease of the central nervous system characterized by inflammatory demyelination and axonal degeneration. It is the commonest cause of permanent disability in young adults. Environmental and genetic factors have been suggested in its etiology. Currently available disease modifying drugs are only effective in controlling inflammation but not prevention of neurodegeneration or accumulation of disability. Search for an effective neuroprotective therapy is at the forefront of multiple sclerosis research.

## 1. Introduction

Multiple sclerosis (MS) is an autoimmune disease of the central nervous system characterised by inflammation, demyelination, and axonal degeneration. Lesions are dispersed in space and time. It is the commonest neurological disease in young adults with resultant permanent disability [1, 2]. There is geographic variation in the incidence and prevalence of MS. In the United Kingdom (UK) annual prevalence and incidence rates are 7/100,000 and 120/100,000 respectively, with an average UK general practice (GP) caring for 2-3 patients in their care. It is common among people of 18–50 years of age [2]. The male to female ratio is about 1 : 3. The higher female preponderance is thought to be due to the effects of hormonal differences that predisposes to a greater environmental susceptibility [3].

## 2. Types of MS

The classic MS is characterised by periods of relapse and remission. A relapse is any attack of demyelination seen clinically as a new or sudden worsening of symptoms lasting longer than 24 hours. Two relapses are usually separated by 30 days and may resolve completely or partially. The first single distinct episode of demyelination in the optic nerves, cerebellum, cerebrum, brainstem, and spinal cord is described as a clinical isolated syndrome (CIS) [4]. It is not MS because there is no dissemination in time and space. The radiologically isolated syndrome (RIS) occurs when there are MRI abnormalities suggestive of demyelination in the absence of clinical correlates [5]. Other types of MS based on the pattern of relapse, remission, and accumulation of disability include relapsing remitting (RRMS), secondary progressive (SPMS), primary progressive (PPMS), and progressive relapsing (PRMS). The likelihood of progressing from CIS to MS is directly related to the presence of CSF oligoclonal bands (OCB), motor symptoms, and finding of high numbers of MRI white matter lesions. Positive MRI white matter lesion at CIS suggest 50% probability of a second relapse within 2 years and 80% within 2 decades, but only 20% of patient with normal MRI will relapse in 20 years [2].

The presence of more than one clinically evident attack of demyelination separated by a period of partial or complete resolution and then stability prior to a second attack defines RRMS. A retrospective diagnosis of benign MS is made when there is lack of significant disability after about 20 years of clinically evident demyelination [6, 7]. In SPMS symptoms gradually worsen without remission. PPMS are marked by a progressively aggressive steady course with little or no recovery from the onset of symptoms. In PRMS, the symptoms steadily get worse from the onset, but with distinct relapse with or without recovery. About 80% of RRMS eventually evolve into SPMS, while about 20% of MS is PPMS.

## 3. Aetiology and Pathogenesis of MS

The aetiology of MS is unclear but epidemiological studies do suggest an environmental and genetic basis, with the lag in environmental exposure before clinical manifestation suggesting a “prodromal phase” [8]. A poorly understood genetic association has been established between MS and human lymphocyte antigen (HLA), particularly HLA-DR2 and HLA-DRB1 [9]. Other genes implicated in MS include interleukin (IL)-7, IL-2, and tumour necrosis factor (TNF) receptor super-family member 1A and interferon regulator factor-8 gene [10]. The genetic basis of the disease is further supported by the fact that certain ethnic groups have a higher chance of having MS such as Native Americans compared with a lower incidence among African Americans [11].

An individual with a genetic susceptibility develops MS when exposed to environmental factors with strong association for developing MS such as Epstein-Barr virus (EBV), smoking, increasing latitude, and vitamin D deficiency. EBV has been documented in 90% of patients with MS [12]. Smokers have a higher risk of developing MS compared to nonsmokers, and this risk is dose dependent [13]. Even in the same region, MS incidence and prevalence increases with latitude and is the strongest risk factor after ethnicity [14]. The relationship between latitude and MS has been thought to be due to the decreasing sunlight and Vitamin D with increasing latitudes, thus establishing an inverse relationship between MS susceptibility and sunlight [15]. The migrant country of origin and the age of migration are also important in MS risk. Migrants after the age of 20 years retain the risk of their home country, while those who migrate earlier adopt the risk of their new country [16].

The exact mechanism underlying the evolution from RIS to CIS and eventually to MS is still unclear. Factors defined by birth such as sex, HLA, and place of birth require the inciting effect of risk factors in the environment to develop clinical MS. MS is thought to be mediated by CD4 and CD8 T-cell and to a lesser extent humoral immune factors. Strong evidence in support of the humoral basis of MS is the intra-CFS synthesis of oligoclonal bands (OCB) [17]. Environmental factors are believed to trigger T-cell autoimmune response against the CNS in the periphery, the activated cells cross the blood brain barrier (BBB) after inflammatory cytokines have disrupted its integrity. The activated T-cells attack myelin-basic protein and myelin oligodendrocytes glycoprotein with antigenic properties similar to that of the inciting environmental factor commonly an infectious agent, a phenomenon referred to as molecular mimicry [18]. The disease is activated by these autoreactive polyclonal lymphocytes and macrophages but propagated by microglial actions and neurodegeneration. The pathological hallmark of MS is inflammation, neurodegeneration, and associated synaptic pathology. Evidence also suggests repair and remyelination by oligodendrocytes. The exact relationship between disease progression marked by the degenerative phase and the immune mechanisms or inflammation is still not clear [19–21]. The possibility of oligodendrocytes apoptosis triggered by viral or glutamate excitotoxicity preceding the inflammatory phase has been observed in some newly formed lesions from neuropathological studies [22]. The resultant inflammation, demyelination, and associated axonal degeneration are responsible for the clinical features of MS.

MS presents a complex picture of the interaction between inflammation and neurodegeneration. Further documented evidence for inflammation includes the production of reactive oxygen species and cytokines and compliment activation, which is believed to initiate myelin damage and secondary axonal loss [23]. The sequence of events between inflammation and neurodegeneration is still hypothetical; some scholars have proposed that neuronal and axonal damage precedes demyelination (inside-out theory), while others think demyelination occurs before axonal damage (outside-in theory) [24].

## 4. Clinical Features of MS

The spatial distribution of MS plaques accounts for its varied clinical features. Optic neuritis one of the commonest presentations of MS manifests as loss of colour vision, decreased visual acuity, and gritty sensation in eye. Other findings in the eye include internuclear ophthalmoplegia, afferent pupillary defect, saccadic ocular pursuit, and acquired pendular nystagmus [25]. Further cranial nerve damage is seen as loss of facial sensation, vertigo, and trigeminal neuralgia. Cortical plaques can result in cognitive impairment affecting memory and attention, but frank dementia is rare. Sensory involvement is commonly seen as numbness, tingling, paraesthesia, tightness, coldness, radicular pain, and itchy sensation.

Spinal plaques result in bladder, bowel, and sexual dysfunction, as well as Lhermitte's sign, which describes as an electric shock-like sensation radiating down the spine on flexion of the neck. Lesions involving the dorsal column result in impaired proprioception. Patient may also have fatigue unrelated to physical activity; Uhthoff's phenomenon where the patient's symptoms worsen with activity that increases body temperature; and features of cerebellar dysfunction such as gait imbalance, dysmetria, decomposition of complex movements, intention tremors, scanning speech, and truncal ataxia. The finding of rubral tremor is an advance brain stem feature in MS, with cardinal features being a complex of ataxia, dysarthria, tremor of the extended upper limb, head titubation, and ophthalmoplegia. There is always a small possibility of another family member having MS but not in an autosomal or mitochondrial pattern of inheritance.

The diagnosis of MS has evolved over the years from the Dawson criteria of 1916, to Schumacher criteria in 1965, superseded by Poser criteria established in 1983 and finally McDonald criteria of 2001. The 2010 revised McDonald criteria for MS diagnosis is based on the presence of 2 or more episodes of symptoms of demyelination at different levels of the CNS separated by at least 30 days or a MRI evidence for dissemination in time and space. In CIS, MS is diagnosed if the MRI at 3 months shows 1 or more gadolinium enhancing lesions. If a second scan is done in CIS at least 30 days from the first and it shows one or more T_2_ lesions on the new scan, a diagnosis of MS can be made. CIS can also be diagnosed by the coexistence of an asymptomatic gadolinium enhancing and nonenhancing lesion at any time. Dissemination in space can be defined clinically by the presence of several episodes affecting difference aspects of the CNS or radiologically by the finding of MRI lesions at more than one level in the CNS. Application of these criteria follows the elimination of other differential diagnoses [26].

The differential diagnosis of MS includes a vast number of clinical conditions [27]. Causes of optic neuritis include Devic's disease; Leber's hereditary optic neuropathy; toxins like tobacco and alcohol; vitamin B-12 deficiency; and other inflammatory disorders like sarcoidosis, vasculitis, and systemic lupus erythemathosus (SLE). Acute disseminated encephalomyelitis (ADEM) is a strong differential of PPMS due to its monophasic attack. Devic's disease is differentiated from RRMS by the presence of aquaporin-4 antibodies and rare involvement of the cerebrum in Devic's disease.

## 5. Investigations

Important investigations would include MRI which is useful for diagnosis, monitoring of treatment, and progression of MS. Lumbar puncture (LP) is done to strengthen the diagnosis of MS by demonstrating the greater presence of OCB in CSF more than that of serum, a finding seen in 95% of patients with MS. CSF OCB is particularly important in the diagnosis of PPMS and in people 50 years and above with nonspecific MRI white matter changes. OCB are also seen in inflammatory conditions like SLE, neurosarcoidosis, Behcet's disease, and Sjögren disease [28]. Visual evoked potential can demonstrate demyelination in the optic nerve even in the absence of any other features of optic neuropathy. Somatosensory evoked potential helps in confirming spinal cord involvement. Other investigations helpful in excluding differentials include Vitamin B12 and folate assay for nutritional deficiencies; antinuclear antibodies, erythrocyte sedimentation rate, and rheumatoid factor for autoimmune and connective tissue diseases; and angiotensin converting enzyme for sarcoidosis.

## 6. Prognosis

There are no clear prognostic guidelines for predicting outcome in MS, but accumulation of disability is closely associated with male sex, older age, patients with PPMS, pyramidal signs, short interattack interval, early residual disability, and presence of brain stem or cerebellar lesion on MRI scan [2]. The absence of CSF oligoclonal bands at the time of diagnosis is a good prognostic factor [7]. After 20 years 50% of MS patients will need a walking device and overall life expectancy is decreased by 10–15 years. Death usually results from secondary infections involving the skin, chest, or urinary tract.

## 7. Treatment of MS

Acute attacks of relapse are treated with oral or intravenous corticosteroids like prednisolone, methylprednisolone, and Dexamethasone. They help to reduce the duration of relapse but have no effect on the long term course of the disease. Plasmapharesis is used if steroids are ineffective. Long term impact on the course of MS is achieved with use of disease modifying therapies (DMT). Some of the disease modifying drugs currently available and their suggested mechanism of action are as summarized in Table 1. Disease outcome is better if DMT is started early, but long term benefit is unclear and data supporting their use in secondary progression is scant [29]. Currently CIS is not treated in the most countries because of the uncertainties regarding efficacy of treatment, the cost of the drugs, and uncertain prognosis of MS at CIS. There is the often puzzling scenario of early immunotherapy having greater benefit if started early in the disease course but also raising the prospect of exposing young nondisabled patients to the well documented toxic effects of these treatments. In countries, where CIS is treated, the approved drugs are interferon (INF)-*β* and glatiramer acetate (GA), which reduces the conversion of CIS to MS over 3 years by about 20%. Symptomatic treatment of other problems of MS is usually undertaken under a multidisciplinary team.

## 8. Licensed Disease Modifying Therapy

Several drugs have been licensed in the European Union (EU) and United States (US) for use as DMT. IFN and GA were the first DMT to be approved for MS. IFN which was first approved in 1997 is currently being marketed as IFN-*β*-1a and IFN-*β*-1b for subcutaneous or intravenous use. The PRISM trial [30] showed a reduction of annualised relapse rate by 27% for patients on IFN-*β*-1a. The side effects of IFN include injection site and hypersensitivity reactions, mood disturbance, liver toxicity, blood disorders, thyroid disease, and flu-like symptoms. Development of neutralising antibodies in about one-third of the patients may affect its efficacy.

GA with a relatively better safety profile was first approved in the EU in 1996. It is given as a daily subcutaneous injection, and the side effects include flu-like symptom, mood disturbances and lipodystrophy. Although the exact mechanisms of action of IFN and GA are unknown, they act by altering the expression of numerous gene products and markers such as major histocompatibility class 1, *β*2-microglobulin, and neopterin. They are currently approved as first line therapy for CIS and RRMS. In the REGARDS study, subcutaneous IFN-*β*-1a or GA given for 96 weeks were assessed for the time to first relapse as primary outcome and number/changes in volume of T2 active lesions as secondary outcome [32]. This well-randomised multicentre study showed that there was no significant difference in both outcomes between the two groups. However, the limitation of this study in its efficacy predictive value might be the use of a population of patients with apparently low disease activity.

Natalizumab is a humanized monoclonal antibody that targets *α*4B1-integrin leading to inhibition of leukocyte migration. The AFFIRM trial which was a well-randomised study reported a reduction of relapse by 68%; progression of disability by 42%; and accumulation of new MRI lesions by 83% at 2 years [33]. Coadministration of Natalizumab with IFN-*β*-1a in the SENTINEL trial was associated with progressive multifocal leukoencephalopathy (PML) prompting its initial withdrawal but was later reintroduced under a new protocol [34]. The new protocol requires the screening of patient for JC virus before being placed on Natalizumab. It was approved in the EU in 2006 for the treatment of evolving severe RRMS as a second line drug administered 4 weekly. The side effects are hypersensitivity reactions, liver toxicity, and increased risk of PML.

Fingolimod is the first introduced oral monotherapy for RRMS in the US and EU, but in England it is currently licensed as a second line drug [35]. It acts by modulating sphingosine-1-phosphate-receptor thus preventing the egress of lymphocytes from lymph nodes [36, 40]. The FREEDOM trial showed that Fingolimod significantly improved annualised relapse rates and MRI end points when compared to IFN and placebo [41]. Must of its benefit including neuroprotective and reparative originated from the TRANSFORMS trials [37, 42]. Its side effects include macular oedema, fatigue, headache, dyspnoea, liver abnormalities, and death from atrioventricular block [43].

Dimethyl fumarate (DF), alemtuzumab, and teriflunomide are still awaiting EU licensing following European Medical Agency (EMA) recommendation. DF acts by activating the nuclear factors resulting in anti-inflammatory, antioxidant, and neuroprotective effects [38]. Its strength of evidence is based on findings from the CONFIRM trial which showed a reduced annualized relapse rate of about 44–53% compared to placebo when given orally to patients with RRMS [44]. Side effects of DF include flushing, gastrointestinal upset, lymphopaenia, and increased liver enzymes.

Teriflunomide a selective immunosuppressant with anti-inflammatory properties inhibits lymphocyte proliferation by blocking dihydroorotate dehydrogenase which is the rate limiting step in pyrimidine synthesis. The TEMSO trial with a large sample size of over 1000 patients showed a 31% reduction in the annualised relapse rate, disability progression particularly at the higher dose, and imaging evidence of reduced disease activity compared with placebo [45]. It is approved for a daily administration in RRMS and potential side effects include hepatotoxicity, diarrhoea, nausea, alopecia, neutropenia, skin rash, and weight loss.

Alemtuzumab is the first humanized monoclonal antibody to be produced. It acts by targeting the surface antigen CD52 expressed on lymphocytes and monocytes resulting in lymphopaenia. It also increases trophic factors involved in neuroprotection. In is approved for intravenous use in adults with active RRMS. Safety concerns include induction of autoimmune diseases like Grave's disease and increased risk of infection.

Mitoxantrone was approved in the US for aggressive or worsening RRMS, SPMS, and PRMS. It acts by inhibition of B-cells, T-cells, and macrophage proliferation [39]. It is cardiotoxic and may predispose to leukaemia. Left ventricular ejection fraction (LVEF) should be measured before Mitoxantrone therapy and is contraindicated if LVEF is less than 50%

## 9. Drugs Undergoing Trials

Future treatments in MS include biologicals currently undergoing trials such as novel monoclonal antibodies, oral immunotherapies, combination immunotherapy, peptide vaccinations, neuroprotective drugs, and reparative strategies using stem cells. The drugs which are currently undergoing trial at different phases for the different types of MS including ATX-MS-1467, daclizumab, ocrelizumab, laquinimod, alemtuzumab, BG-12, and teriflunomide for RRMS; masitinib, natalizumab, and Siponimod for SPMS; masitinib and ocrelizumab for PPMS [46]. Currently, laquinimod is undergoing licensing in the UK while alemtuzumab, BG-12, and teriflunomide are undergoing NHS appraisal.

Current areas of research in animal models that could be subsequently tried in humans include neuroprotective agents like glutamate antagonist (riluzole), sodium channel blockers (flecainide), cannabinoids receptor antagonist, statins, and erythropoietin, some of which have already been brought into clinical trials [47]. Among the currently investigated therapies statins seem to hold a lot of promise in MS. The neuroprotective properties of statins, which decrease cholesterol production by blocking the enzyme 3-hydroxy-3-methylglutaryl coenzyme A (HMG-C0 A), have already been reported in Alzheimer's disease. Animal studies have also reported a similar benefit in MS [48–50]. The exact mechanism of neuroprotection is unclear, but it is believed to be due to statins' ability to inhibit immune mediated inflammatory response by binding to *β*2 integrin on leukocyte. This action is specific to lovastatin, simvastatin, and mevastatin but not pravastatin [51]. Another possible mechanism of action is by promoting the release of neurotrophic factors involved in neurogenesis and synaptogenesis [52, 53].

Following damage to axons and glial cells, enhancing their repair is fundamental to the future of MS care. In this regard the potential benefit of stem cell is the focus of most ongoing trials [54]. Current data suggest that beyond repair, neural stem cells may actually enhance* in situ* release of immunomodulatory molecules [55]. The challenge to stem cell therapy however still remains ethical issues and technical difficulties in drug delivery.

## 10. Costs of Disease Modifying Therapy

Several reports have documented the high cost of DMT to private and public payers for this therapy. The concept of “risk sharing scheme” was introduced in 2002 to monitor the long term cost effectiveness of DMT on a cohort of about 5000 MS patients in the UK using the Expanded Disability Status Score (EDSS) scale. The enabling circular mandates the NHS trust and DMT manufacturers to jointly finance the scheme towards DMT procurement, MS infrastructural development, and staff training. The drugs and their manufacturers are Avonex (IFN-*β*-1a), Biogen Idec Inc.; Betaferon (IFN-*β*-1b), Bayer Schering Pharma; Copaxone (glatiramer acetate), Teva/sanofi Aventis; and Rebif (IFN-*β*-1a), Merck Serono [51, 56].

## 11. Conclusion

Although the currently available DMT are effective in controlling inflammation, they are disappointing in controlling the most important aspect of MS responsible for accumulation of disability being the progressive phase of the disease. There is therefore a need for the shift in the focus of treatment from inflammation to degeneration in preventing accrual of disability [50]. Cure for MS has been defined in terms of halting the progress of the disease, reversal of neurological deficit, and development of a preventive scheme [51]. The prevention of MS at the suggested prodromal phase when environmental factors exact their effect could be an opportune time to reverse MS early in its cause. This can be done in part by instituting preventive measures against environmental agents, such as infections, and promoting smoking cessation [8]. Reversal of disease progression and repair of damaged nerves lie in the realm of stem cell therapy, but this has not been helped by the heterogeneity of MS as a disease and lack of exact animal models needed for cutting edge studies and development of appropriate therapies with ability to alter the long term outcome or even prevent the evolution of MS.

## Figures and Tables

**Table 1:** Summary of some disease modifying drugs used in multiple sclerosis.

Drug	Approved indication(s)	Possible mechanism of action	Some common adverse effects	Route(s)	References
IFN-*β*-1a	RRMS, CIS	Inhibition of CD4+ T-cells and enhancement of CD8+ T-cells.	Hypersensitivity reaction, hepatotoxicity, haematologic disorders, and injection site reactions.	Subcut./IM	[30, 31]

IFN-*β*-1b	RRMS, Progressive MS	The same as above.	Same as above.	The same as above.	[30, 31]

Glatiramer acetate	RRMS, CIS	Downregulates the expression of autoreactive T-cells.	Injection site reaction, mood disturbance, and hypersensitivity reaction.	Subcut.	[31, 32]

Natalizumab	RRMS, Severe Remitting MS	Acts on *α*4 integrins resulting in inhibition of leukocyte migration into the CNS.	Increased risk of PML, hepatotoxicity, and hypersensitivity reaction.	IV	[33, 34]

Fingolimod	RRMS after IFN	Modulates sphingosine-1-phosphate receptors preventing the egress of lymphocytes from lymph nodes.	Hepatotoxicity, atrioventricular block, increased risk of malignancy, and mood disturbance.	Orally	[35–37]

Dimethyl fumarate	Relapsing MS	Activate nuclear factors resulting in anti-inflammatory, antioxidant, and neuroprotective properties.	Increase hepatic enzymes, gastrointestinal upset, and lymphopaenia.	Orally	[38, 39]

Mitoxantrone	Aggressive MS, SPMS	Inhibits B-cell, T-cell, and macrophage proliferation.	Cardiotoxicity, Leukopaenia,	IV	[39]

(Im: intramuscularly; iv: intravenously; subcut.: subcutaneously).
